# Detection and monitoring of *Drosophila suzukii* in raspberry and cherry orchards with volatile organic compounds in the USA and Europe

**DOI:** 10.1038/s41598-021-85884-1

**Published:** 2021-03-25

**Authors:** Nicholas R. Larson, Jaime Strickland, Vonnie D. Shields, Antonio Biondi, Lucia Zappalà, Carmelo Cavallaro, Stefano Colazza, Lucía-Adriana Escudero-Colomar, Felix Briem, Heidrun Vogt, François Debias, Patricia Gibert, Emmanuel Desouhant, Aijun Zhang

**Affiliations:** 1 Invasive Insect Biocontrol and Behavior Laboratory, Beltsville Agricultural Research Center-West, USDA-ARS, Beltsville, MD 20705 USA; 2 Department of Biological Sciences, Towson University, Towson, MD 21252 USA; 3 Department of Agriculture, Food and Environment, University of Catania, 95123 Catania, Italy; 4 Department of Agriculture, Food and Forest Sciences, University of Palermo, Viale delle Scienze, 90128 Palermo, Italy; 5 Sustainable Plant Protection, IIRTA, Mas Badia, Canet de la Tallada S/N, 17134 Girona, Mas Badia Spain; 6 Federal Research Centre for Cultivated Plants, Institute for Plant Protection in Fruit Crops and Viticulture, Julius Kühn Institute (JKI), Schwabenheimer Straße 101, 69221 Dossenheim, Germany; 7 Federal Research Centre for Cultivated Plants, Institute for Biological Control, Julius Kühn Institute (JKI), Heinrichstraße 243, 64287 Darmstadt, Germany; 8 CNRS, UMR5558 LBBE, Univ Lyon, Université Claude Bernard Lyon 1, 69622 Villeurbanne, France

**Keywords:** Ecology, Zoology, Ecology

## Abstract

Spotted wing drosophila (SWD) causes significant economic loss in fruit crops to growers worldwide. There is immediate need for efficacious and selective monitoring tools that can detect infestations early. Previously, volatile organic compounds derived from apple were studied and a quinary chemical component blend (QB) was identified as the key SWD attractant in a blueberry orchard in the United States. This study’s aim was to determine whether previously observed QB efficacy, selectivity, and early detection levels could be attained within raspberry and cherry fields in the USA and Europe. Results demonstrated that sticky trap baited QB dispenser provided earlier SWD detection potential than the usually adopted apple cider vinegar (ACV) trap. The number of SWD captured/trap by QB baited trapping systems was significantly lower than that of the ACV trap. However, percent SWD/trap of QB baited traps was same within cherry. Lower non-target capture will save farmer/grower’s labor and time allocated to traps installation and drosophila species identification. Within the USA, SWD selectivity of QB baited liquid traps was consistently greater than sticky trap in raspberry field, suggesting that the QB dispenser can be an alternative to the standard ACV lure and that trap design could improve selectivity further.

## Introduction

Worldwide *Drosophila suzukii* Matsumura (Diptera: Drosophilidae), or spotted wing drosophila (SWD), is a significant pest that causes large economic losses in thin-skinned fruits^1–6^. Significant efforts have been directed into the experimentation and implementation of reliable and sustainable control^7–10^ and monitoring tools for SWD, much of which has been focused on chemical lure development^11–17^. In addition to lure development, trap development has been explored by altering trap color, shape, and size^18–22^. Recently traps with tunnel entries to prevent escape and pesticide coated surfaces to increase mortality of SWD have been tested/developed^23^. However, even with the advancements in trapping systems there is still a significant need to further improve SWD monitoring especially when it comes to the chemical lures^16,22,24–27^.

To continue advancing chemical lures for SWD, the attractive properties of five volatile organic compounds (acetoin, ethyl octanoate, phenethyl alcohol, ethyl acetate, and acetic acid) were identified as the key attractive compounds from fresh and fermenting apple juices^16^. A controlled-release dispenser, for emission control of this quinary chemical component blend (QB), was developed to increase the longevity of the lure in a subsequent study^28^. They determined that the selectivity of these components when formulated into a dispenser approached 50% (percent of SWD captured compared to total capture). Further work found a *ca.* threefold decrease in non-target drosophilid captures, within a wooded area near a blueberry field^28^. This significant non-target drosophilid activity reduction is needed to reduce the amount of work required for identification of the target pest^29–31^, in addition to overall non-target reduction through lure development ultimately reducing the ecological toll from capturing non-target insects in general^13^.

Furthermore, landscape variation can impact pest populations^32^, with evidence showing that higher SWD populations can be found in areas having more diverse habitats^33^. In this context, the objective of this current study was to examine the efficacy, selectivity, and early pest detection of a QB chemical dispenser within raspberry and cherry fruits across five countries: the United States, France, Germany, Italy, and Spain. The landscape differences between the various locations will provide much needed capture data on how well this SWD trap system performs when deployed in these regions. With the primary goal to provide evidence of a successful versatile lure, which can be deployed for SWD monitoring trap capture campaigns.

## Results

### Spotted wing drosophila captures within the United States

**Figure 1:**
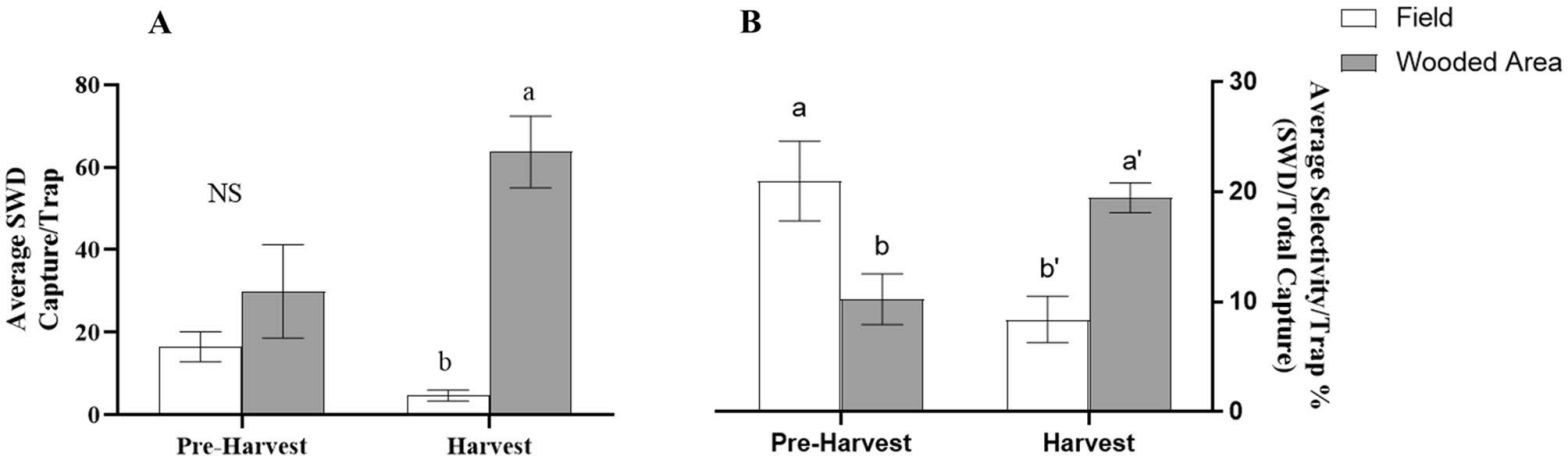
Average capture (**A**) and selectivity (**B**) per dry sticky trap baited with QB during pre-harvest and harvest periods during 2019 in a raspberry field and nearby wooded area within the United States. QB data have been adjusted by the capture amounts in the control dry sticky traps. Means have been separated by two-way ANOVA followed by a Tukey’s post hoc test. Different letters above bars (within harvest periods) indicate significant differences (α = 0.05). Bars represent standard error of the mean.

**Figure 2:**
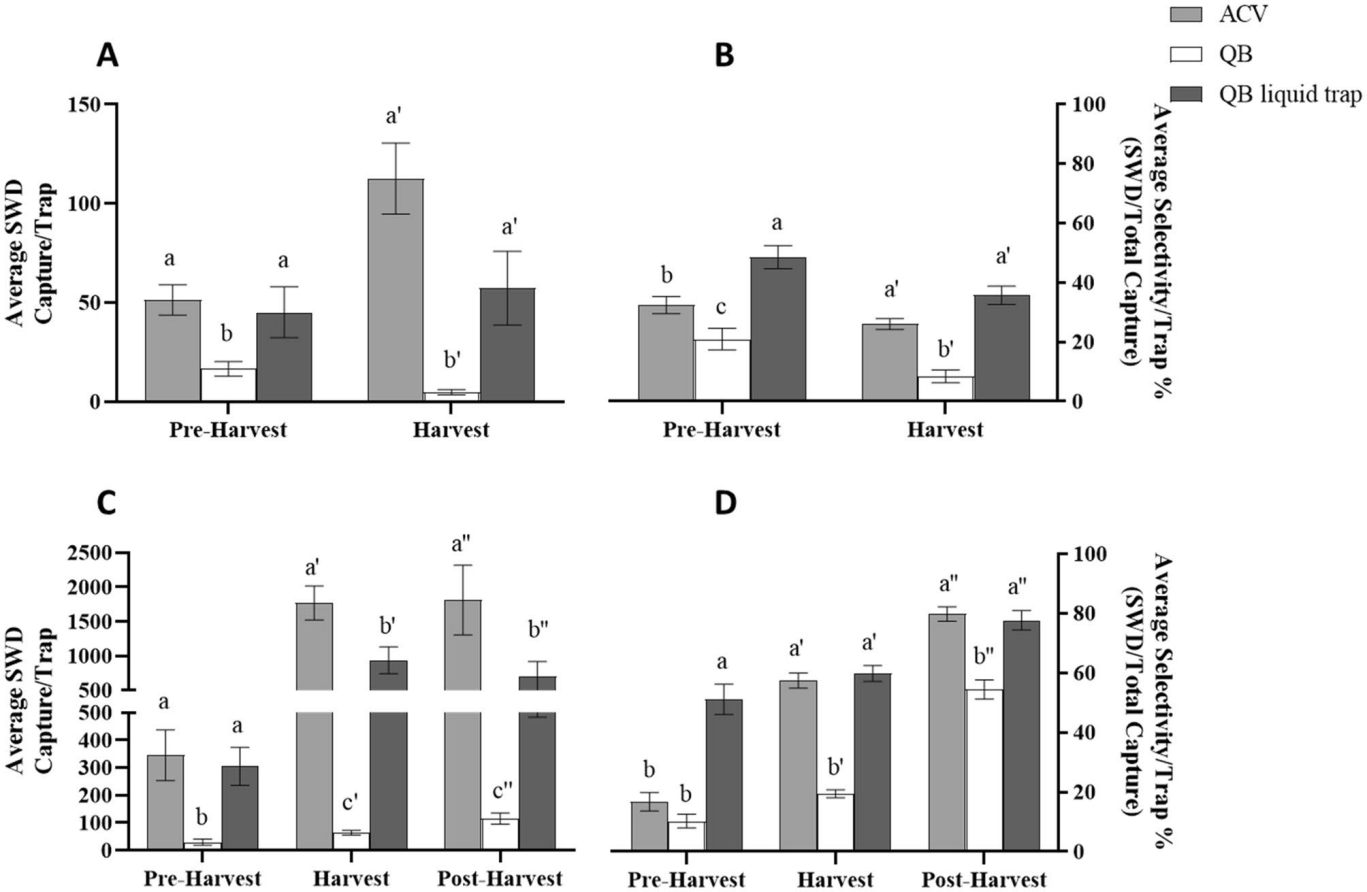
Average capture and selectivity per trap in a raspberry field (**A**,**B**) and wooded area (**C**,**D**) within the United States. QB represent dry sticky traps baited with quinary blend controlled-release sachets. QB Wet Trap. QB data has been adjusted by the capture amounts in the control traps. Means have been separated by One-Way ANOVA with a Tukey’s post hoc test. Different letters above bars (within harvest periods) indicate significant differences (α = 0.05). Bars represent standard error of the mean.

Comparison between the raspberry field and wooded area, during pre-harvest and harvest periods to account for presence of developing and fully ripened fruit, SWD captures and selectivity per QB dry sticky trap is found in Fig. 1A,B. No difference was found in average capture per trap between either area during the pre-harvest period, nor was there a difference between these and the field during the harvest period. The wooded area during the harvest period captured the greatest amount of SWD/trap (*F*_1,209_ = 7.335, *P* = 0.007) (Fig. 1A). Dry sticky traps baited with QB had a significantly higher selectivity during the pre-harvest period in the raspberry field than in the wooded area but was not significantly different from the trap selectivity in the wooded area during the harvest period. The pre-harvest wooded area trap selectivity was not different from the harvest field trap selectivity. While the harvest field trap selectivity was lower than that of the wooded area trap selectivity during the same period (*F*_1,203_ = 23.6, *P* < 0.0001) (Fig. 1B). Average SWD capture per trap and average selectivity per trap within a raspberry field and nearby wooded area in Maryland, can be found within Fig. 2A–D. In the raspberry field, dry sticky traps baited with QB had the lowest capture (Pre-harvest: *F*_2,82_ = 13.94, *P* < 0.0001; Harvest: *F*_2,139_ = 62.22, *P* < 0.0001) and selectivity (Pre-harvest: *F*_2,81_ = 12.83, *P* < 0.0001; Harvest: *F*_2,137_ = 46.46, *P* < 0.0001) rates compared to the other two liquid systems in both the pre-harvest and harvest periods (Fig. 2A,B). Liquid traps baited with QB had higher selectivity than ACV during the pre-harvest period. Within the wooded area dry sticky traps baited with QB had the lowest capture (Pre-harvest: *F*_2,122_ = 26.36, *P* < 0.0001; Harvest: *F*_2,172_ = 110.8, *P* < 0.0001; Post-harvest: *F*_2,54_ = 10.97, *P* < 0.0001) and selectivity (Pre-harvest: *F*_2,120_ = 22.79, *P* < 0.0001; Harvest: *F*_2,169_ = 108.6, *P* < 0.0001; Post-harvest: *F*_2,54_ = 23.79, *P* < 0.0001) rates in all trapping periods except with the exception of pre-harvest where ACV baited traps had a similar selectivity. During pre-harvest QB baited liquid traps had the highest selectivity, but then in the remaining trapping periods selectivity rates between ACV and QB baited liquid traps did not differ. Average weekly capture and selectivity for both the raspberry field and wooded area can be found in Fig. S1A–D.

### First capture of spotted wing drosophila captures within the United States

Within the United States the ACV baited liquid trap and QB baited dry sticky trapping systems caught male SWD during the first week of deployment in the wooded area (Table1). Traps were placed on May 7, 2019 and were picked up on May 14, 2019 for processing. Within the raspberry field ACV liquid traps were initially deployed on the ground on May 7, 2019, as the raspberry bushes were not developed enough to support hanging traps. The QB baited dry sticky traps were not deployed until June 4, 2019. Once placed the QB dry sticky traps captured a single SWD male compared to the ACV trapping eight SWD males. Liquid traps baited with QB were placed on June 19, 2019 and immediately captured SWD males and females during the first week of trapping.

**Table 1:** Week, date, and number of males and females of first capture of *D. suzukii* by different traps within a raspberry field and a nearby wooded area in the United States in 2019.

Date	United States											
	Raspberry Field						Wooded Area					
	QB^α^		QB-LT		ACV		QB		QB-LT		ACV	
	♂	♀^†^	♂	♀	♂	♀	♂	♀^†^	♂	♀	♂	♀
7 May	–	–	–	–	Traps Placed		Traps Placed				Traps Placed	
14 May	–	–	–	–	–	–	1	–	–	–	22	–
22 May	–	–	–	–	–	20	–	–	–	–	–	174
29 may	–	–	–	–	–	–	–	–	–	–	–	–
4 June	Traps Placed		–	–	–	–	–	–	–	–	–	–
12 June	1	–	–	–	8	–	–	–	–	–	–	–
19 June	–	–	Traps Placed		–	–	–	–	Traps Placed		–	–
26 June	–	–	75	86	–	–	–	–	1107	230		–

QB are sticky traps baited with USDA QB, QB-LT are liquid Victor traps baited with USDA QB, and ACV are traps baited with apple cider vinegar. ^†^(On female symbol in table): Due to the difficulty in detecting female SWD on sticky traps female counts were not performed. ^α^dry sticky traps in the USA could not be placed into field until raspberry bushes were tall enough to allow for them to hang.

### Comparison of QB baited dry sticky traps in France, Germany, Italy, and Spain

**Figure 3:**
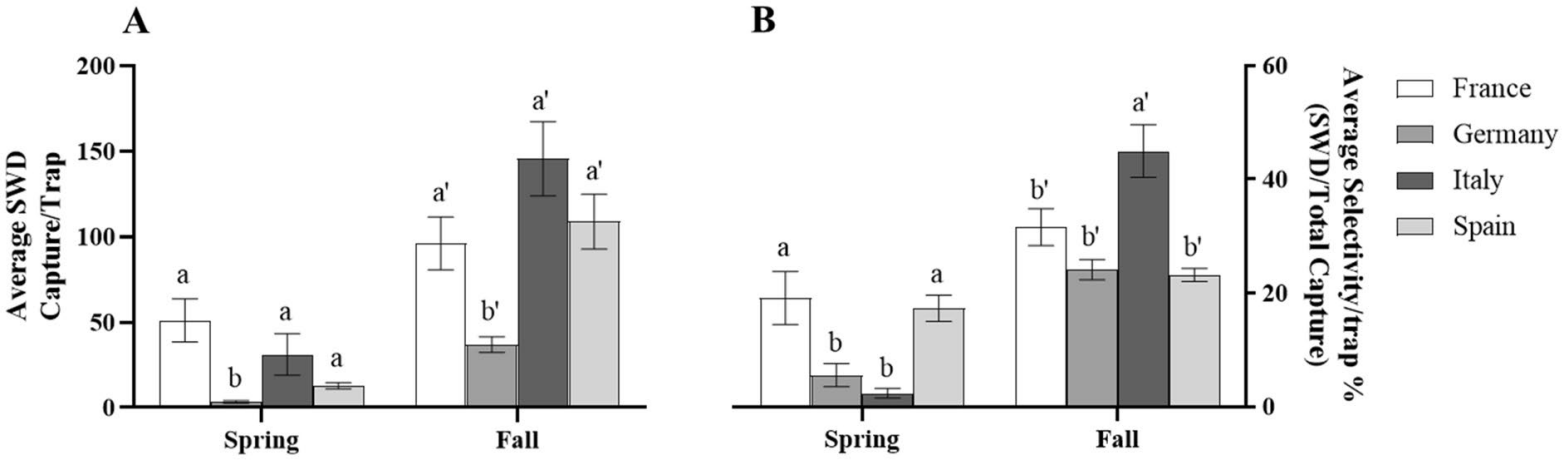
Average capture (**A**) and average selectivity (**B**) per trap of QB baited dry sticky trap systems during the spring and fall within France, Germany, Italy, and Spain. Bars represent standard error of the mean. Means separated by One-way ANOVA with a Tukey’s post hoc test. Different letters above bars (within season) indicate significant differences (α = 0.05). Data represented are untransformed.

**Figure 4:**
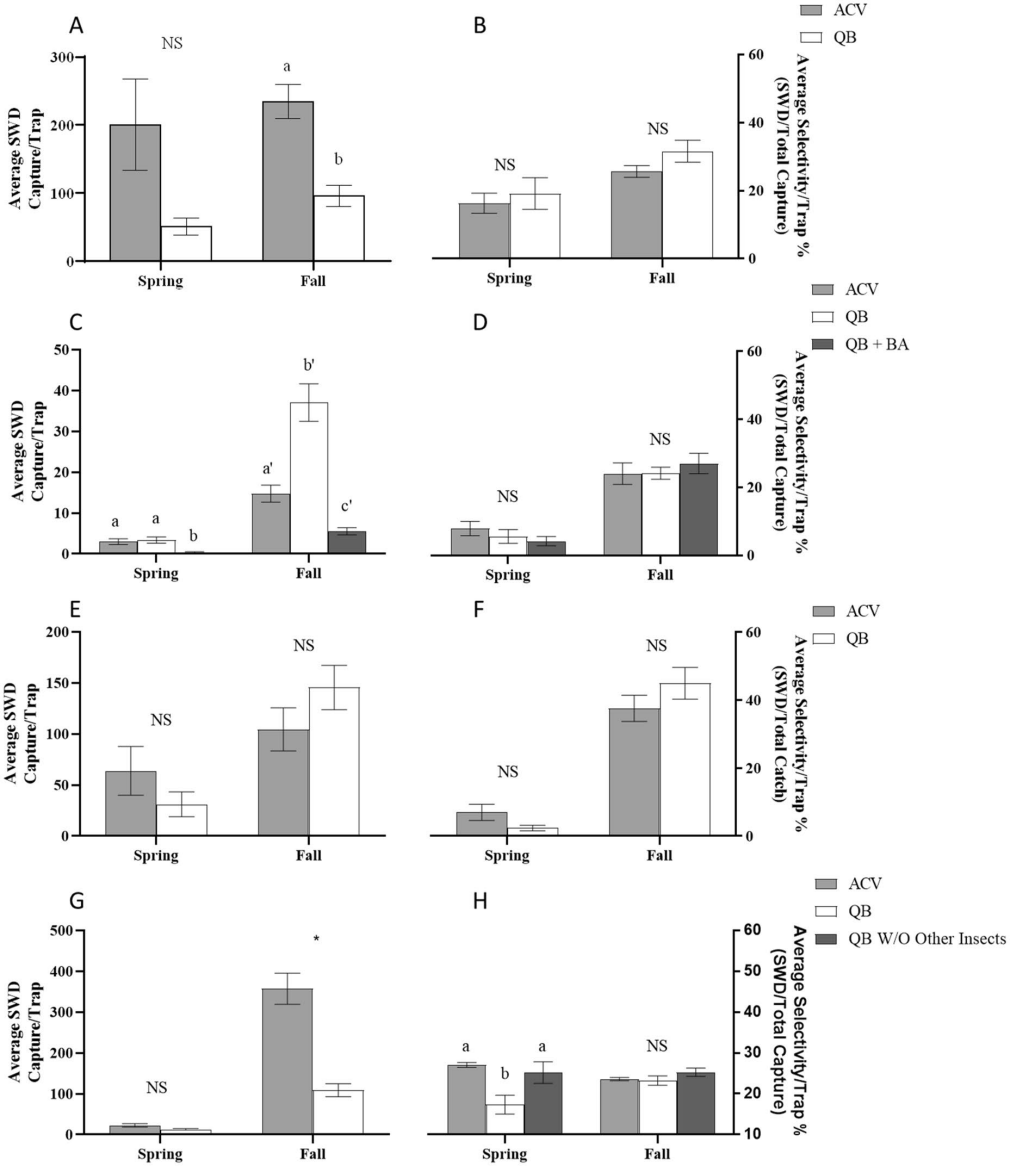
Average capture and selectivity per trap in cherry trees within France (**A**,**B**), Germany (**C**,**D**), Italy (**E**,**F**), and Spain (**G**,**H**). ACV represents liquid traps baited with apple cider vinegar. QB represent dry sticky traps baited with quinary blend controlled-release sachets. QB data has been adjusted by the capture amounts in the control traps. QB + BA represents traps baited with the quinary blend controlled-release sachets and boric acid within a liquid trap. QB w/o other insects represents the quinary blend controlled-release sachets without the other insect data included to calculate selectivity due to the ACV liquid trap capturing zero “other insects”. Means have been compared by an unpaired t-test. Different letters above bars (within season) indicate significant differences (α = 0.05). Bars represent standard error of the mean. NS above bars indicate there is no significant difference between the means.

A comparison of the QB baited dry sticky traps average SWD captures and selectivity from France, Germany, Italy, and Spain can be found within Fig. 3. Germany had significantly lower average trap captures compared to the other countries within both trapping periods, while the remaining countries showed no difference between each other during the same periods. (Spring: *F*_3,147_ = 18.8, *P* < 0.0001; Fall: *F*_3,128_ = 10.5, *P* < 0.0001. B) (Fig. 4A). Germany and Italy had significantly lower average selectivity per trap ratios compared to the ratios of France and Spain during the spring trapping period (Spring: *F*_3,147_ = 18.8, *P* < 0.0001) (Fig. 4B). However, during the fall the average selectivity ratio for traps within Italy increased to be significantly greater than the other selectivity ratios from the other countries (*F*_3,128_ = 10.5, *P* < 0.0001).

### Spotted wing drosophila captures within France

Sticky traps baited with QB dispenser typically caught lower numbers of SWD per trap on average (Fig. 4A) compared to ACV baited traps (Spring: *t* = 1.25, *df* = 70, *P* = 0.22; Fall: *t* = 5.33, *df* = 62, *P* < 0.0001), however the selectivity of dry sticky traps baited with QB dispenser was not significantly different from ACV baited traps (Spring: *t* = 0.54, *df* = 70, *P* = 0.59; Fall: *t* = 1.64, *df* = 62, *P* = 0.12) (Fig. 4B). Trapping averages during the fall period, when the cherry fruits were absent, showed a small increase in the number of SWD caught per trap in both ACV and QB baited system, though only the average capture for the QB dispenser baited system significantly increased compared to spring capture (*t* = 4.52, *df* = 78, *P* < 0.0001). The average number of males caught per trap baited with QB dispenser nearly doubled during fall, however, this was still significantly lower than the average number caught by traps baited with ACV. The selectivity for the trapping systems during the spring trapping period was relatively low and not significantly different. The selectivity of the two trapping systems increased significantly during the fall trapping period compared to the spring trapping period (QB: *t* = 2.14, *df* = 66, *P* = 0.036; ACV: *t* = 2.63, *df* = 66, *P* = 0.011), however they again were not significantly different from each other. Average weekly capture and selectivity for France can be found in Fig. S2A,B.

### Spotted wing drosophila captures within Germany

In the spring there was not a significant difference between ACV baited traps and the QB baited dry sticky traps in the average number of SWD caught per trap. However, there was a significant difference between those trapping systems and liquid traps baited with the QB dispenser with boric acid (QB + BA) added to the liquid solution (*F*_2,162_ = 10.72, *P* < 0.0001) (Fig. 4C). All trapping systems saw an increase in the average number of SWD caught per trap during the fall trapping period from the spring period. Traps baited with QB saw a 11-fold increase (*F*_54,39_ = 1.86, *P* < 0.0001) in average capture which made it significantly greater than the average capture in ACV baited traps that only increased by 4.9-fold (*F*_54,39_ = 1.97, *P* < 0.0001). JKI QB traps baited with QB + BA saw a 14-fold increase in capture during the fall (*F*_39,54_ = 2.7, *P* < 0.0001), however, the average capture continued to be significantly lower than either of the other trapping systems (*F*_2,117_ = 59.54, *P* < 0.0001). As with the results from France, there was no significant difference in selectivity between the trapping systems during the spring or fall trapping periods (Spring: *F*_2,159_ = 1.868, *P* = 0.1579; Fall: *F*_2,117_ = 0.3514, *P* = 0.7044) (Fig. 4D). However, each system did see an increase in selectivity from spring to fall. ACV baited traps increased three-fold (*F*_54,39_ = 1.16, *P* < 0.0001), QB baited dry sticky traps increased four-fold (*F*_54,39_ = 3.58, *P* < 0.0001), and QB + BA baited traps increased six-fold (*F*_39,52_ = 1.4, *P* < 0.0001). Average weekly capture and selectivity for Germany can be found in Fig. S2C,D.

### Spotted wing drosophila captures within Italy

Data collected from cherry trees in Italy show that there was statistically no difference between ACV baited liquid traps and the QB baited dry sticky traps in average number of SWD caught per trap (Spring: *t* = 0.362, *df* = 38, *P* = 0.72; Fall: *t* = 1.49, *df* = 38, *P* = 0.26) or selectivity per trap (Spring: *t* = 0.36, *df* = 38, *P* = 0.15; Fall: *t* = 0.71, *df* = 38, *P* = 0.48) in the spring or fall (Fig. 4E,F). During the spring trapping period traps baited with QB caught on average half of the amount of SWD individuals that traps baited with ACV caught. During the fall trapping period, average capture for ACV baited traps increased by 1.6-fold per trap (*t* = 3.66, *df* = 38, *P* = 0.0008), while the average capture of QB baited dry sticky traps increased by 4.7-fold (*t* = 5.47, *df* = 38, *P* < 0.0001). Selectivity of traps baited with QB was about three-fold lower than the selectivity of traps baited with ACV in the spring, however the values were not significantly different. Both trapping systems saw an increase in selectivity from the spring trapping period to the fall trapping period. Traps baited with ACV had an average selectivity increase of 5.4-fold (*t* = 7.4, *df* = 38, *P* < 0.0001) while traps baited with QB had a selectivity increase 19-fold (*t* = 10.78, *df* = 38, *P* < 0.0001). Average weekly capture and selectivity for Italy can be found in Fig. S2E,F.

### Spotted wing drosophila captures within Spain

Trapping within cherry trees in Spain revealed that there was not a significant difference in average trap capture between ACV baited traps and QB baited dry sticky traps in the spring (*t* = 1.47, *df* = 78, *P* = 0.15) (Fig. 4G). There was a significant difference found between the two systems within the fall trapping period with ACV baited traps capturing 3.2-fold more SWD than traps baited with QB (*t* = 6.38, *df* = 78, *P* < 0.0001). The selectivity of traps baited with ACV during the spring trapping period was 3.3-fold greater than traps baited with QB (*F*_2,117_ = 8.165, *P* < 0.0001) (Fig. 4H). However, as the ACV baited capture zero “other insects” likely due to trap design, adjusting the selectivity of the QB traps by removing the “other insects” captured from the analysis will provide an idea of what could be achieved utilizing a trap system similar to what was used for the ACV lure. This increases the selectivity of the QB baited dry sticky traps by 1.5-fold making the selectivity value not significantly different from that of the ACV baited traps. The selectivity of the trapping systems during the fall trapping period were not significantly different (*F*_2,117_ = 1.405, *P* = 0.2495). Comparing the selectivity between the spring and fall trapping periods the selectivity of QB baited dry sticky traps was increased by 1.3-fold (*F*_39,39_ = 4.19, *P* = 0.0075), while the selectivity of ACV baited traps decreased slightly (*F*_39,39_ = 2.16, *P* < 0.0001). The adjusted value of the QB traps remained approximately the same between trapping periods (*F*_39,39_ = 6.85, *P* = 0.71). Average weekly capture and selectivity for Spain can be found in Fig. S2G,H.

### First capture spotted wing drosophila captures within France, Germany, Italy, and Spain

First capture, for the European data, are found within Table 2. In Spain, France, and Italy both trapping systems caught male and female SWD within the first week of deployment. Traps placed in Germany however found that the QB baited dry sticky traps caught SWD a week earlier than the ACV baited liquid traps. Traps placed in Germany were deployed on April 30, 2019 and first detection of male SWD was made by the QB baited dry sticky traps at the May 7, 2019 collection day. ACV baited traps did not detect male SWD until June 11, 2019, five weeks after the first male detection made by the QB baited dry sticky traps. ACV liquid traps had the first female detection a week later than the male detection by the QB liquid trap, i.e., on May 14, 2019 collection day. The QB baited dry sticky traps did not have a female detection until May 28, 2019 collection, 2 weeks after the ACV traps. The modified JKI QB traps had their first female SWD detection on June 25, 2019, three weeks after the QB baited dry sticky traps had their first female detection, and five weeks after the ACV baited traps. These traps additionally did not capture any male SWD until the fall trapping period.

**Table 2:** Week, date, and number of males and females of first capture of *D. suzukii* by different traps within Cherry orchards in Spain, Germany, France, and Italy in 2019.

First Catch 2019 in Cherry Trees																		
Date	Spain				Germany						France				Italy			
	QB		ACV		QB		JKI QB		ACV		QB		ACV^§^		QB		ACV	
	♂	♀	♂	♀	♂	♀	♂^‡^	♀	♂	♀	♂	♀^†^	♂	♀	♂	♀	♂	♀
19 April	Traps Placed		Traps Placed		–	–	–	–	–	–	–	–	–	–	–	–	–	–
26 April	37	101	15	305	Traps Placed		Traps Placed		Traps Placed		Traps Placed		Traps Placed		–	–	–	–
7 May	–	–	–	–	3	–	–	–	–	–	135	–	61	155	–	–	–	–
14 May	–	–	–	–	–	–	–	–	–	1	–	–	–	–	Traps Placed		Traps Placed	
23 May	–	–	–	–	–	–	–	–	–	–	–	–	–	–	15	1	2	8
28 May	–	–	–	–	–	2	-	–	–	–	–	–	–	–	-	–	–	–
3 June	–	–	–	–	–	–	–	–	–	–	–	–	–	–	–	–	–	–
11 June	–	–	–	–	–	-	–	–	1	–	–	–	–	–	–	–	–	–
25 June	–	–	–	–	–	–	–	3	–	–	–	–	–	–	–	–	–	–

QB are sticky traps baited with USDA quinary blend, JKI QB are traps baited with USDA QB blend + 200 ml tap water and 1% boric acid, and ACV are traps baited with apple cider vinegar. †Due to the difficulty in detecting female SWD on sticky traps female counts were not performed in France. ‡Male SWD were not caught by JKI QB traps in Germany until a sampling period that began in the fall. §France ACV was a 1:1:1 mix of ACV, red wine, and water.

## Discussion

In the spring, summer, and fall of 2019 field trapping trials were conducted in five countries with the aim to screen the efficacy, selectivity, and early detection of a QB dispenser previously developed for SWD monitoring within raspberry and cherry. Results from the current study show that trapping systems utilizing a sticky trap as the capture apparatus, baited with the QB dispenser regularly underperformed, compared to ACV baited liquid traps, in number of SWD caught per trap, in both raspberry and cherry fruits. Further, the selectivity of the sticky trap baited with QB dispenser trapping system was significantly lower than ACV in the raspberry field and wooded area within USA. Though, there was typically no difference between the selectivity of ACV baited liquid traps and the QB baited dry sticky traps within cherry hosts. The main result relies on the fact that liquid traps baited with the QB dispenser exceeded the selectivity of the ACV trapping system during the sampling period prior the fruit harvest in both the raspberry field and wooded area in the USA. The QB baited trapping systems captured SWD at the same time as the ACV traps in Spain, France, and Italy, where the SWD population was already established in the fields at the time of trap development. In Germany, where SWD occurred later and the QB sticky trap system captured SWD males a week before ACV baited traps. This suggests that the QB dispenser is a viable option to be a component of a trapping system, which does not utilize a dry sticky trap, for SWD within raspberry and cherry fruits.

The quinary blend, utilized within this study, was first reported to achieve a selectivity of 47% within blueberry hosts during fruit abundance in 2018^16^. Within this current study the average selectivity for the QB dispenser coupled with the sticky trap ranged from 2 to 19% during the period when host fruit was abundant, and 21–45% when host fruit was absent. This was shown in this study with QB baited liquid traps having selectivity averages of *ca.* 49% pre-harvest when host fruit was not abundant and *ca.* 36% during harvest when host fruit was abundant. The difference between trapping systems may in part be explained by the simplistic nature of the sticky trap as it utilizes a glue-like substance, found on the outside of the trap, to capture individual insects. This allows for insects that are drawn toward it by the lure to get stuck to the trap, however this can also lead to accidental captures.

Host odors are another significant barrier to overcome to achieve efficient and selective trapping systems. This is demonstrated by the observation that trapping during off-season regularly resulted in increased selectivity for all trapping systems. While non-target capture did increase for both the QB and ACV trapping systems, the ACV trapping system showed an increase that was three-fold greater than what was seen in the QB trapping system. Further the results from trapping within the wooded area within the USA lend support that there is a need to overcome host odor abundance. Trap selectivity within this area ranged from 10 to 78% across each type of trapping system, again increasing over time for each trapping system. There was a decrease in non-target capture over time with a corresponding increase in SWD captures for each trapping system within the wooded area. The decrease in non-target capture can be partially explained by the fact that over time as the weather becomes colder during the later season the number of non-targets available will decrease. Further, SWD captures can remain the same or increase as SWD has a winter morph that help it survive during the harsher cold months^34,35^. However, the selectivity of the QB baited liquid trapping system never dropped below 50% during each of the trapping periods. Suggesting that even during the warmer periods, when there would be potentially wild hosts within the wooded area, the QB dispenser continued to perform well. This could suggest that the dispenser is outcompeting the wild hosts within the wooded area due to a potential lower abundance of hosts resulting in a lower amount of host odor for the lure to compete with. Compare this to a field of raspberry host fruit or cherry orchard where host odor would likely be abundant and ubiquitous resulting in more competition for the lure to overcome.

The present data suggest that liquid traps baited with a selective lure would be ideal to address these issues, however there is a downside to utilizing complex traps. In addition to the amount of labor required for deployment and maintenance of liquid traps, processing times of the catch are also increased compared to sticky traps^36^. However, development of a trapping system that has been designed to provide accurate early detection would benefit farmers/growers by giving them a tool to quickly and easily determine when to deploy SWD remediation efforts. The QB baited dry sticky trap has been shown here to capture SWD at the same time as the ACV baited liquid traps within cherry orchards in France, Italy, Spain, and within the wooded area in the USA because the SWD population have been established already in the fields. Within Germany the sticky trapping system detected SWD a week earlier than the ACV trap and six weeks earlier than the JKI QB liquid traps. Although all traps were deployed at the same time in Spain, Germany, and France, SWD infestation was much delayed in the tested cherry orchards in 2019 in Germany, therefore, early SWD detection capability was observed. In addition, Larson et al.^28^ found that the sticky trap baited QB blend detected SWD two to five weeks earlier than ACV traps, indicating that this trapping system could detect SWD population earlier than the ACV trap and provide farmers/growers quick and easy assessment tool for timely intervention in orchard, if traps were set up early enough.

To conclude this study has shown that the QB dispenser can be a utilized as a successful detection tool when coupled with either a sticky trap or liquid trap in two different crop systems and various regional settings. In addition to the previous studies showing that the dispenser can also be utilized within blueberry, there is a significant opportunity to further test additional SWD non-raspberry and non-cherry hosts. While the overall efficacy and selectivity of the sticky trapping system was either below or equal to that of the liquid trapping systems, the practical use of the trap would likely be for just a short period (i.e., until first detection). Utilization of the system would not be advised for long periods of trapping due to significant off-target captures. Once first detection is made control efforts can be initiated and the traps removed. Further, regional areas that would not allow the use of sticky traps due to beneficial capture effects could choose to utilize the liquid trap system baited with the QB dispenser. There is opportunity to further increase the efficacy and selectivity of this trapping system though modification of the trap itself. SWD have been shown to be attracted to darker red and black colors^22,37,38^. Combining color attractants in addition to physical exclusion structures within the trap (i.e., entry tunnels^23^ or smaller entrance holes), such as with the traps that were tested in France and Germany, could provide a significant integrated pest management tool for farmers/growers to utilize to manage this significant pest. Prior to acceptance by growers/producers a cost benefit analysis would need to be done to ensure that this new trapping system would provide substantial benefit.

## Methods

### Chemicals

The chemicals used in this study were purchased from Sigma-Aldrich (St. Louis, MO, USA): 3-hydroxy-2-butanone, also known as acetoin (AT), 99%, CAS 512-86-0; ethyl octanoate (EO), 99 + %, CAS 106-32-1; acetic acid (AA), 99.7 + %, CAS 64-19-7; phenethyl alcohol (PE), 99 + %, CAS 60-12-8; and ethyl acetate (EA), solvent grade, CAS 141-78-6.

### Controlled-release dispensers with dry sticky traps

Methodology for dispenser construction has been fully described in Larson et al.^28^. In summary, SWD controlled-release dispensers were made of clear polyethylene tubing, consisting of four sachets, each sachet contained EA (5 mL), PE (1 mL), AA (5 mL), and AT/EO (2 mL 1:1 v/v ratio). For placement into the traps, one of each of the sachets (EA, PE, AA, and AT/EO) were tied together by a paper clip and secured onto the non-sticky side of a dry sticky trap (single sided 406.4 × 120.7 mm window bug captures, Alpha Scents, Inc., West Linn, OR) with a twist-tie. Dry sticky traps without dispenser were placed within the field to serve as blank controls.

### Experimental sites/orchards and exposure of traps

Field tests were conducted at several locations within five countries: the United States, France, Germany, Italy, and Spain. For all locations spring indicates that host fruits are developing/ripened, while a fall indicates trapping periods where host fruits are absent. A summary of location, trap type, and date of deployment can be found in Supplementary Table S1.

In the United States, field trials were conducted within a managed (fungal treatments) raspberry field and nearby non-managed wooded area located at Butler’s Orchard (22222 Davis Mill Rd, Germantown, MD, USA) during the summer and fall of 2019 and 4 types of traps were used. On June 12, 2019, dry sticky traps (baited with QB dispenser), blank control dry sticky traps (without dispenser; hereafter called control), and apple cider vinegar (called ACV traps) liquid traps (Victor Poison Free Yellow Jacket & Flying Insect Trap, Woodstream Co., Lancaster, PA, USA) were set up in the prior mentioned raspberry field and wooded area (N = 5 replicates for each treatment). Liquid traps (baited with QB controlled-release dispenser) were deployed into the field and wooded area on June 26, 2019. Victor liquid traps contain four holes in the lid of the trap measuring *ca.* 10 mm in diameter for insect access. After a dry sticky trap was hung in the field (mid-upper canopy), the paper layer was torn off to expose the sticky layer. All traps were collected every 7 days. The contents of the ACV and QB liquid traps were emptied into separate containers and returned to the laboratory for identification. Dry sticky traps were collected, the dispensers removed, and the traps were folded in half with the sticky side in taking care to not press the trap together fully so that the capture could be counted within the laboratory. Liquid traps were then refilled, and the dry sticky trap dispensers were placed onto new dry sticky traps. Controlled-release sachets containing EA were changed every other week, all sachets were changed out every four weeks from start. The raspberry field sampling concluded on October 16, 2019 signaling the end of the harvest for the crop. The wooded area was continually sampled weekly through December 4, 2019.

Field tests in France began on May 1, 2019 and were conducted in two sites separated by 30 km. The first site was a protected garden within the Claude Bernard University Lyon 1. The site consisted of two ponds, bushes, and several wild fruit trees including cherry. The second site was located within a private garden in Chalamont that consisted of various fruit trees including cherry, apple, and fig. Both gardens received no chemical treatments. Within each site two areas were chosen to place traps (3 types of traps were used; see Table S1). The areas within the Lyon site were separated by 75 m, while the areas within the Chalamont site were separated by 30 m. In each area, three trees were selected for trap placement. Each tree was separated from the others by a minimum of 5 m. Trap placement was randomized within the three selected trees weekly in each area. Each area received one of each of the previously mentioned traps for a total three traps per area (six per site). The ACV trap was a 1:1:1 mixture of apple cider vinegar, red wine, and water. The container was a 1-L, red, translucent plastic bottle that had twenty-five 2 mm diameter holes drilled randomly into the bottle for insect access. Sampling ended on July 2, 2019, and then was begun again starting September 2, 2019 through October 29, 2019 to sample during a period when host fruits were not available in the sampled location.

Field tests within Germany began on April 30, 2019 and were conducted in the experimental field at the Institute for Plant Protection in Fruit Crops and Viticulture of the Julius Kühn Institut (JKI) in Dossenheim. Four types of traps were utilized in this field trial. The two dry sticky trap systems with and without QB, and two types of liquid traps. For liquid traps, the JKI-trap was used which had 21 small (2.5 mm diameter) entry holes^39^. The traps contained either 200 ml cloudy ACV bait, comprising of ACV with 5% acetic acid content, diluted to 40% with tap water and 0.025% odorless detergent^39^, or 200 ml tap water with 1% boric acid and 0.025% odorless detergent with the controlled release dispenser containing the QB bait suspended above the water/underneath the lid (JKI QB). Five traps of each type were placed into a cherry orchard (0.64 ha; variety: Regina) in a randomized complete block design and collected weekly. During the sampling period, the cherry orchard was typically treated with fungicide and herbicide, but only once for insect infestation (aphids) with Pirimicarb (Pirimor, Syngenta) on May 15, 2019. Specific treatments against *D. suzukii* were not performed. The traps were separated by seven trees each. All traps were replaced every seven days. The dispensers were removed from the collected traps and placed onto the new sticky and liquid traps. The cups of the liquid traps were cleaned and reused in the following week, after identifying all captured individuals. Sampling during the host fruit season ended on July 16, 2019, two weeks after cherries were ripe. In the experimental orchard, the cherries were not harvested. Sampling was then started back up during non-fruiting season and when daily mean temperatures dropped for the first time below 10 °C (Fig. “October” see end of file), beginning October 1, 2019 and ending November 19, 2019.

Field tests within Italy began on May 16, 2019 and were conducted in commercial cherry orchards (no chemical treatments used) located on the East slope of the volcano Etna, in Sant’Alfio (Catania—Sicily). Three types of trap systems were tested in this location, the two dry sticky trap systems with and without QB as described previously, and a homemade liquid ACV bait consisting of 75% apple cider vinegar, 24.5% red wine, and 0.5% sugarcane. The trap itself was made from a 500 ml plastic water bottle. The opening to the bottle was covered with a plastic net (0.1 × 0.6 mm openings) to allow entry to only smaller insects. One of each of the trapping systems was placed into each of four locations that were separated by 100 to 400 m to each other. Trap captures were collected weekly and controlled release dispensers were changed as previously described. Initial sampling during fruit abundance ended on June 19, 2019, and then began again during the non-fruiting season on September 19, 2019 and concluded on October 23, 2019.

Field tests within Spain began on April 26, 2019 and were conducted within a commercial cherry orchard located at Ventalló municipality in the Girona province (NE of Spain) and controlled by the Institute for Research and Technology in Food and Agriculture (IRTA) in Girona. In the spring Spinosad and deltamethrin treatments were applied to the orchard. No treatments were applied in the fall. Three types of traps were tested in this location, the sticky trap without the controlled-release QB dispenser, the sticky trap with the controlled-release QB dispenser, and the liquid ACV bait trap in a Contech (Contech Enterprises Inc., Victoria, BC, Canada) 2013 model trap. The trap contained three holes for insect entrance, one 2 mm diameter hole in the lid, and two 6 mm diameter holes in the sides of the body of the trap. Five replicates of each trap were placed a minimum of 5 m apart and controlled release dispensers were replaced as previously described. Sampling during fruiting season ended July 12, 2019, and then began during the non-fruiting season on October 24, 2019 and completed on November 11, 2019.

### Trap capture counting

Captures from traps were sorted into the following categories: SWD (*D. suzukii*—male and female), African fig fly [(*Zaprionus indianus* Gupta (Diptera: Drosophilidae)], other drosophilids, big flies (house flies, crane flies, etc.), or other arthropods. SWD individuals are morphologically identifiable by the wing spots at the tips of the wings and by the presence of a black sexual comb in the forelegs for males, and the distinguishable sclerotized and serrated ovipositor found within the females^40,41^, that is used to cut the skin of healthy host fruits for oviposition^42,43^. Trap captures from both types of liquid traps were strained using a sieve to separate out the trap capture from liquid. The strained individuals were then placed into a Petri dish and separated and counted under microscopes. In the USA, captures containing greater than four milliliters of strained individuals were approximated by counting two milliliters, and then multiplying the counts by the calculated factor. Dry sticky trap captures were visually counted under a microscope. Due to the difficulty in discerning the female serrated ovipositor (degradation occurred), the USA counts for female SWD were approximated by calculating the percentage of SWD females caught in the liquid traps and then using that percentage to adjust the total counts from the sticky trap.

### Statistical analyses

Within the USA capture data were used to compare a managed raspberry field and a nearby non-managed wooded area. Within the European countries capture data were pooled from each test location to compare differences between countries within cherry fruits. Attractiveness (# *D. suzukii* caught) per trap and selectivity (% *D. suzukii*/total captures) per trap were utilized to compare the lure/trap systems. The average weekly capture and selectivity were used to determine capture trends over time. Trap data were Log(x + 1) transformed to conform the assumptions of normality more closely. Percentage of SWD captured on traps (i.e., selectivity) were arcsine-square-root transformed to achieve distribution normality. Transformed data were utilized to compare means. Means of capture results were compared using a One-way followed by a Tukey’s post hoc test, or a student’s t-test to determine significance (α = 0.5). A Two-way ANOVA followed by a Tukey’s post hoc test was utilized to compare the mean SWD capture and selectivity between the raspberry field, wooded area, and period within the USA. Untransformed data are presented in figures. Control dry sticky trap captures were subtracted from the QB dry sticky trap captures to adjust for the captures made by control traps. Statistical analysis was performed using GraphPad Prism (GraphPad Software V9, LLC San Diego, CA, USA).

## Supplementary Information

Supplementary Figure S1.

Supplementary Figure S2.

Supplementary Table S1.
