# Supplementary material for: Detection and monitoring of Drosophila suzukii in raspberry and cherry orchards with volatile organic compounds in the USA and Europe
Source: Sci Rep. 2021 Mar 25;11:6860. doi: 10.1038/s41598-021-85884-1 (PMC7994672; doi:10.1038/s41598-021-85884-1)
Supplement: Supplementary file 1 — Supplementary Figure S1. [file 41598_2021_85884_MOESM1_ESM.docx]

For Scientific Reports

**Detection and Monitoring of *Drosophila suzukii* in Raspberry and Cherry Orchards with Volatile Organic Compounds in the USA and Europe**

**Nicholas R. Larson^1,2^, Jaime Strickland^1,2^,** **Vonnie D. Shields^2^,** **Antonio Biondi^3^, Lucia Zappalà^3^, Carmelo Cavallaro^3^, Stefano Colazza^4^, L. Adriana Escudero Colomar^5^, Felix Briem^6,7^, Heidrun Vogt^6^,** **François Debias^8^, Patricia Gibert^8^, Emmanuel Desouhant^8^ & Aijun Zhang^1^**

^1^Invasive Insect Biocontrol and Behavior Laboratory, Beltsville Agricultural Research Center-West, USDA-ARS, Beltsville, MD 20705, USA

^2^Department of Biological Sciences, Towson University, Towson, MD 21252, USA

^3^Department of Agriculture, Food and Environment, University of Catania, 95123, Catania, Italy

^4^Department of Agriculture, Food and Forest Sciences, University of Palermo, Viale delle Scienze, Palermo, 90128, Italy

^5^Sustainable Plant Protection, IRTA, Mas Badia, Canet de la Tallada S/N, 17134, Girona, Spain

^6^Julius Kühn Institute (JKI) – Federal Research Centre for Cultivated Plants, Institute for Plant Protection in Fruit Crops and Viticulture, Schwabenheimer Straße 101, 69221 Dossenheim, Germany

^7^Julius Kühn Institute (JKI) – Federal Research Centre for Cultivated Plants, Institute for Biological Control, Heinrichstraße 243, 64287 Darmstadt, Germany

^8^Univ Lyon, Université Claude Bernard Lyon 1, CNRS, UMR5558 LBBE, Villeurbanne, F-69622, France

Author for correspondence:

Aijun Zhang

e-mail: aijun.[zhang@ars.usda.gov](mailto:zhang@ars.usda.gov)

Mention of trade names or commercial products in this publication is solely for the purpose of providing specific information and does not imply recommendation or endorsement by the USDA. USDA is an equal opportunity provider and employer.


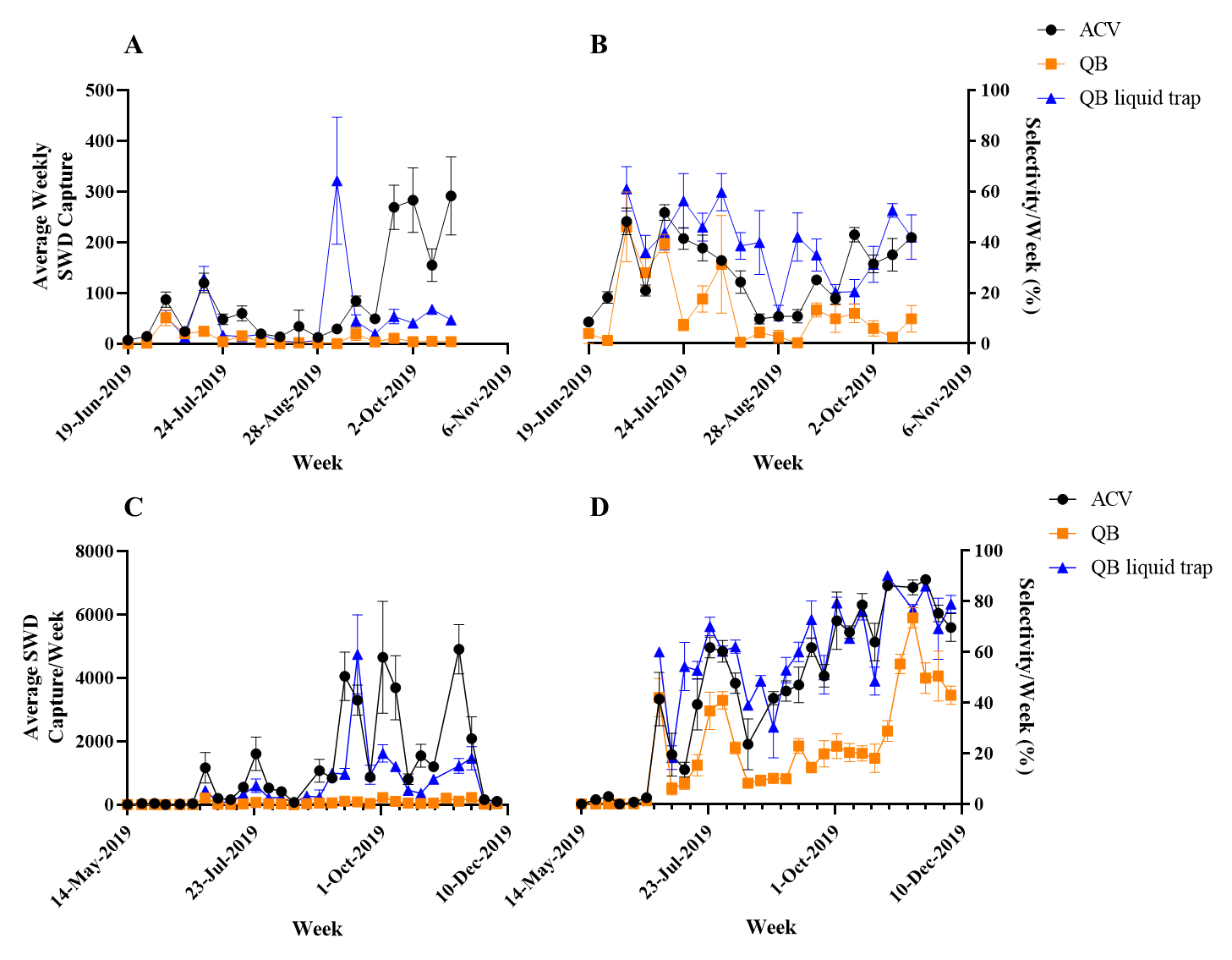


**Figure 1S**. Average capture and selectivity per week in a raspberry field (A & B) and wooded area (C & D) within the United States.
